# Threshold for neural tube defect risk by accumulated singleton loss-of-function variants

**DOI:** 10.1038/s41422-018-0061-3

**Published:** 2018-07-05

**Authors:** Zhongzhong Chen, Yunping Lei, Yufang Zheng, Vanessa Aguiar-Pulido, M. Elizabeth Ross, Rui Peng, Li Jin, Ting Zhang, Richard H. Finnell, Hongyan Wang

**Affiliations:** 1 Obstetrics and Gynecology Hospital, State Key Laboratory of Genetic Engineering at School of Life Sciences, Institute of Reproduction and Development, Fudan University, Shanghai, 200011 China; 2 Key Laboratory of Reproduction Regulation of NPFPC, Collaborative Innovation Center of Genetics and Development, Fudan University, Shanghai, 200032 China; 3 Departments of Molecular and Cellular Biology and Medicine, Baylor College of Medicine, Houston, TX 77030 USA; 4 Collaborative Innovation Center for Genetics & Development, School of Life Sciences, Fudan University, Shanghai, 200438 China; 5 Institute of Developmental Biology & Molecular Medicine, Fudan University, Shanghai, 200433 China; 6 Center for Neurogenetics, Weill Cornell Medicine, New York, NY 10021 USA; 7 Capital Institute of Pediatrics, Beijing, 100020 China; 8 Children’s Hospital, Fudan University, 399 Wanyuan Road, Shanghai, 201102 China; 9 Institutes of Biomedical Sciences, Fudan University, Shanghai, 200032 China

Dear Editor,

Neural tube defects (NTDs) are a class of major structural malformations affecting the brain and spinal cord. They are among the most common congenital anomalies with a worldwide prevalence of 0.1%.^1,2^ Elucidating the genetic basis of their complex etiology has eluded our best efforts to date. Although there are more than 400 genes capable of producing an NTD phenotype when mutated in the mouse,^3,4^ studies of human candidate genes based on mouse NTD genes have not been informative, except for genes in the planar cell polarity pathway.^5^ Recently, an omnigenic model of inheritance was proposed for complex traits, suggesting that the associated signals tend to spread across almost the entire genome.^6^ In light of this new perspective on the genomic architecture of complex traits, we re-evaluated whole-genome sequencing (WGS) data from three different NTD cohorts (Han Chinese, Caucasian USA, and Middle Eastern/Qatar).

We initially evaluated our Chinese NTD cohort (100 cases, primarily anencephaly, Supplementary information, Table S1) and used the 1000 Genomes Project (1KGP)^7^ as controls (208 Chinese Han). Due to the limited sample size of the NTD cohort and uneven coverage of 1KGP sequences (higher coverage in coding regions), only rare (MAF < 0.01) protein-coding variants in NTDs and controls were selected for functional prediction. The selected deleterious missense (D-mis) and loss-of-function (LoF) variants were further compared with 1KGP and ExAC databases (MAF_1KGP_ < 0.001 and MAF_ExAC_ < 0.001). Although D-mis and LoF variants are considered more likely to be causative variants, we failed to observe more rare D-mis variants in our Chinese NTD cases compared to the 1KGP controls (one-sided Wilcoxon test, *P* = 1; Fig. 1a).

**Fig. 1:**
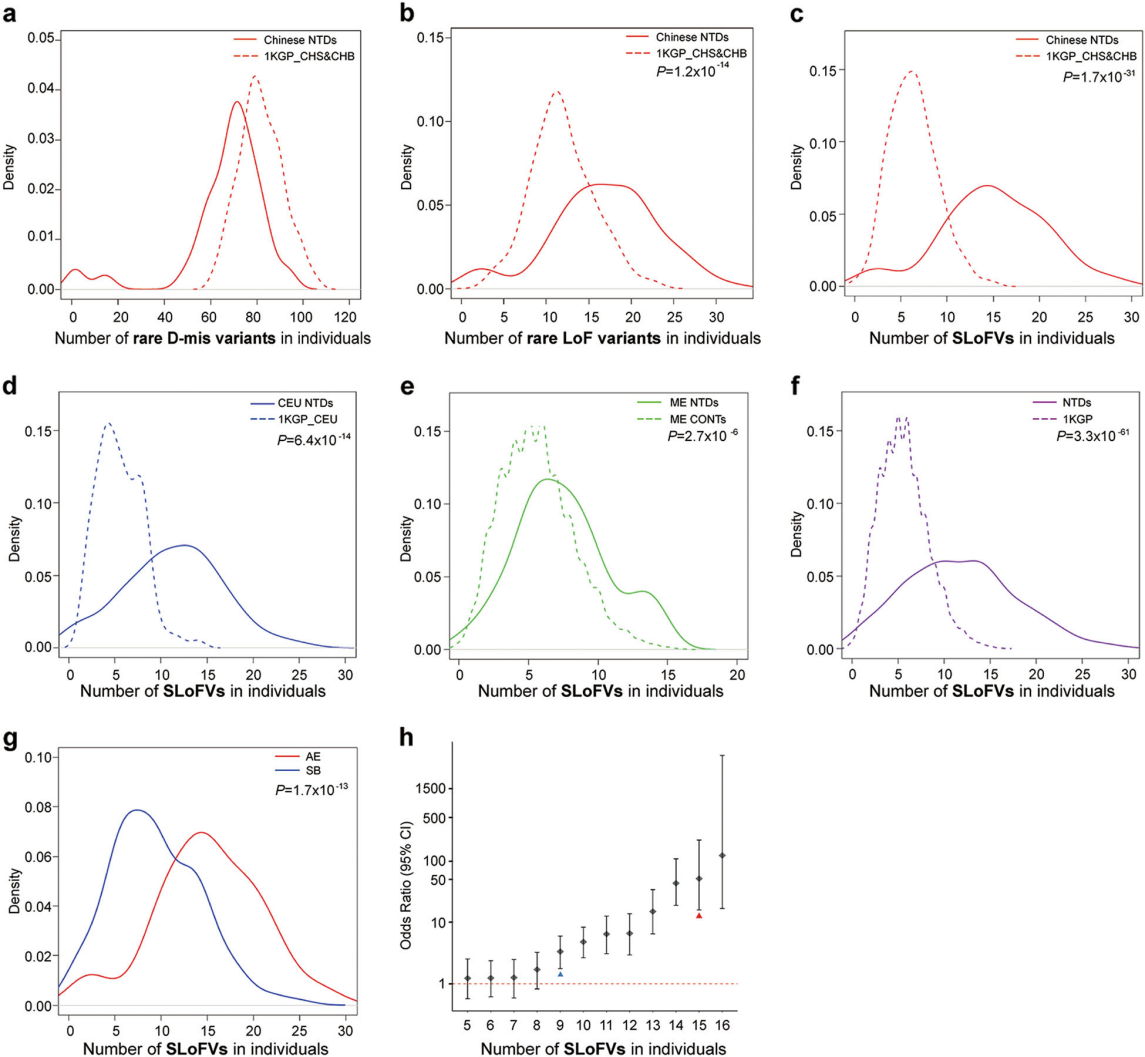
**a** Chinese NTD cases do not carry more rare D-mis variants than controls from the 1KGP. **b** The distribution of rare LoF variants shows significantly more LoF variants for Chinese NTDs than controls. **c**–**e** Significantly more SLoFVs in NTD samples than their matched controls for Chinese (**c**), Caucasian in USA (**d**), and Middle Eastern (ME) cohorts (**e**). **f** Combined data shows significantly more SLoFVs for NTDs than controls from 1KGP. **g** Significantly more SLoFVs were found in anencephalic (AE) than spina bifida (SB) samples. **h** Odds ratios of SLoFVs in NTDs. The blue arrowhead represents the median number of SLoFVs in SB and the red arrowhead represents the median number of SLoFVs in AE. The density (*y*-axis) at each point is the average contribution from each of the kernels at that point

We also did not observe significant enrichment of damaging variants in human orthologs of 249 mouse NTD-associated genes^4^ (*χ*^2^ test, *P* = 0.48; Supplementary information, Table S2). By contrast, when only the rare LoF variants were examined, there are significantly more such variants in NTD cases (median = 17) than in controls (median = 12; two-sided Wilcoxon test, *P* = 1.2 × 10^–14^; Fig. 1b). This suggested that rare LoF variants statistically correlate well with NTDs.

We wanted to validate our observations in different cohorts; however, when we examined WGS data from the 1KGP, we found that the number of rare LoF variants varies among different populations. However, the number of singleton LoF variants (SLoFVs), which are those LoF variants that appeared only once when compared to entire 1KGP data, is very similar among different populations.^7^ This suggests that the number of SLoFVs is a stable and reliable genomic indicator of NTD risk in humans. We re-examined the SLoFVs in our Chinese cohort and found that the median number of SLoFVs per NTD case is 15, whereas the medium number of SLoFVs is 6 in controls (two-sided Wilcoxon test, *P* = 1.7 × 10^–31^; Fig. 1c). We went on to examine the SLoFVs in our US and Middle Eastern NTD cohorts. Seventy-four US spina bifida samples were compared to 99 Caucasians from the 1KGP. Again, a statistically significant difference was detected, with 11.5 SLoFVs in NTDs and 5 SLoFVs in controls (two-sided Wilcoxon test, *P* = 6.4 × 10^–14^; Fig. 1d). The WGS data from a Middle Eastern cohort consisting of 69 spina bifida samples and 108 matched controls (no matched controls in 1KGP) were also examined, and a statistically significant difference was detected, with 7 SLoFVs in NTDs and 5 SLoFVs in controls (two-sided Wilcoxon test, *P* = 2.7 × 10^–6^; Fig. 1e). When the three NTD cohorts (243 cases) were combined and compared to 2,504 controls from the 1KGP, the median number of SLoFVs was 11.5 in NTDs and 5 in controls (two-sided Wilcoxon test, *P* = 3.3 × 10^–61^, Fig. 1f; Binomial test, *P* = 5.97 × 10^–237^, Supplementary information, Table S3). Both results reached a Bonferroni-corrected threshold based on correction for testing of ~20,389 genes (*P* < 2.5 × 10^–6^).

Further comparison between the two major subtypes of NTDs, anencephaly (from Chinese cohort) vs. spina bifida (from US cohort) demonstrated that anencephalic cases carried more SLoFVs than spina bifida cases (15 vs. 9, two-sided Wilcoxon test, *P* = 1.7 × 10^–13^; Fig. 1g). This suggested that the more severe the subtype of NTDs, the more SLoFVs it likely contains. Therefore, we calculated the odds ratios (ORs) of NTDs with different numbers of SLoFVs. Our results demonstrated that there is a threshold SLoFV number for NTD risk. When the number of SLoFVs reaches 9, the OR for NTD is >1 [OR = 3.3 (1.8–6.0)] (Fig. 1h). When an individual carries more SLoFVs, the risk for NTD increases exponentially, e.g., OR = 4.8 (2.7–8.3) for 10 SLoFVs, OR = 6.6 (2.9–13.8) for 12 SLoFVs, and OR = 43.1 (18.8–107.6) for 14 SLoFVs.

Both gene and pathway distributions of those SLoFVs identified in NTD cases from Chinese Han and US CEU cohorts were analyzed to determine whether there is any specific enrichment. Each SLoFV was mapped to KEGG pathways; the frequency of pathways with mutations, based on the percentage of individual NTD cases carrying mutations in each pathway, was calculated. There are 14 pathways containing SLoFVs in > 10% of the NTD samples (Supplementary information, Fig. S1), including the PI3K-AKT signaling pathway, tight junction, and focal adhesion pathways. Some of these pathways, such as tight junctions, were also found to differ significantly at the transcriptome level between NTD and control fetal cells isolated from amniotic fluid^8^ (Supplementary information, Fig. S2). However, there is no pathway in NTDs that is significantly enriched with SLoFVs after Bonferroni correction (Supplementary information, Table S4). In fact, the expression of over 20,000 genes was detected in NTD fetal cells from amniotic fluid,^8^ which suggests that most of the genome is transcriptionally active during neural tube development.

We have now demonstrated that the genetic basis for NTD risk can be measured by the accumulation of SLoFVs in one’s genome. Approximately nine SLoFVs is a genomic threshold for NTD risk, regardless of their genetic background or ethnicity. Together with the finding that no single pathway was enriched for SLoFVs in NTD cases, our study suggests that almost all genes have some minor impact on the etiology of NTDs. In summary, our data demonstrated how a complex reproductive disadvantage disease like NTDs can benefit from the omnigenic model.^6^ This enhances our understanding of the genomic architecture underlying susceptibility to these severe congenital malformations.

Materials and Methods are available in Supplementary information, Data S1.

## Electronic supplementary material

Supplementary information, Data S1

Supplementary information, Tables S1-S4

Supplementary information, Figures S1 and S2
