# Supplementary material for: Threshold for neural tube defect risk by accumulated singleton loss-of-function variants
Source: Cell Res. 2018 Jul 5;28(10):1039–41. doi: 10.1038/s41422-018-0061-3 (PMC6170406; doi:10.1038/s41422-018-0061-3)
Supplement: Supplementary file 1 — Supplementary information, Data S1 [file 41422_2018_61_MOESM1_ESM.pdf]

Supplementary information, Data S1

Material and Methods

Human subjects

Three NTD cohorts (Supplementary information, Table S1), including 100 Chinese Han, 74 Caucasian in Texas USA, and 69 Middle Eastern (ME) samples were used. The studies described herein were conducted in accordance with the Declaration of Helsinki ethical principles for human research. Protocols followed were reviewed and approved by the local ethics committees prior of the study. Written informed consent from the parents or guardians of the children was obtained for all subjects.

Muscle samples of aborted fetuses from 100 Chinese Han NTDs (primarily anencephaly, mean age 20.7 ± 4.7 gestation weeks, 60% female) were collected between 2004 and 2013 in Shanxi province, China.

Saliva samples were collected using a DNA collection kit (ORAcollect for Pediatrics (OC-175), DNAgenoTek) from 74 US Caucasian NTDs (non-syndromic myelomeningocele, mean age 7.5 ± 5.4 years, 51.4% female), which were collected between 2008 and 2013 from Texas, USA. Patients enrolled at the Dell Children's Medical Center of Central Texas (DCMCCT). Genomic DNA of each test subject was isolated using the Gentra Puregene Kit (Qiagen) and was quantified using a NanoDrop2000 (Thermo Scientific).

Blood samples from 69 ME NTDs (non-syndromic myelomeningocele, mean 7.23 years, 59& female) and 108 ME controls were collected and DNA was extracted using Qiagen chemistry (QIAmp, Qiagen #51194). Genomic DNA was quantified using a NanoDrop2000 and DNA quality was assessed on a Bioanalyzer 2100 (Agilent).

WGS data of 2054 healthy individuals from the 1KGP[1] was also used, which includes 208 Chinese Han population (CHS + CHB) and 99 CEU (Utah residents with ancestry from northern and western Europe) in USA.

Illumina sequencing

Two independent sequencing studies were performed. Chinese NTDs were sequenced on an Illumina X10 platform, while the CEU and ME samples were sequenced on a HiSeq2500 platform. The average depth of coverage is 30× for all samples.

Read mapping and variant annotation

The Chinese NTD fastq files were mapped to the hg38 reference sequence using the Burrows-Wheeler Aligner (BWA)[2]. We applied GATK[3] base quality score recalibration, indel realignment, duplicate removal, and performed SNV (SNP and INDEL) discovery and genotyping across all samples simultaneously using standard hard filtering parameters or variant quality score recalibration according to GATK

Best Practices recommendations [4,5]. The variants considered in this analysis are restricted to GATK 'PASS' variants. The CEU and ME sequenced data was processed based on the CASAVA pipeline (1.9.0a1, Illumina) using hg19 as the human reference genome. Variants were further filtered by high genotype quality (≥ 20) in each sample. Coordinates of variants were converted between hg19 and hg38 using CrossMap[6].

Variants (SNVs and Indels) were annotated with information from Ensembl release 84 using the Variant Effect Predictor (VEP)[7] and Ensembl canonical and APPRIS[8] transcripts were utilized. Each variant was classified into groups of LoF (loss of function, including splice acceptor/donor, stop gained/lost, initiator codon and frameshift indels)[9], missense, synonymous and others based on the Sequence Ontology (SO)[10]. The functional impacts of missense mutations were also predicted using Polymorphism Phenotyping version 2 (Polyphen2)[11] and Sorting Intolerant From Tolerant (SIFT)[12] via VEP[7]. The missense variants, that are predicted to be damaging by PolyPhen-2 and deleterious by SIFT, were annotated as deleterious mutations (D-mis).

Rare damaging variant analysis

Minor allele frequency (MAF) of variants was calculated based on each cohort. Only rare (MAF < 0.01) protein-coding variants in NTDs and controls were selected for functional predictions. The selected damaging (LoF and D-mis) variants in both

cases and controls were further compared with data in the 1KGP (1000 Genomes Project) and ExAC databases (http://exac.broadinstitute.org). The variants with $MAF_{1KGP} < 0.001$ and $MAF_{ExAC} < 0.001$ were selected for analysis. D-mis are the missense variants which are predicted to be damaging by PolyPhen-2 and deleterious by SIFT. LoF (loss of function), includes splice acceptor/donor, stop gained/lost, initiator codon and frame-shift indels. We also evaluated singleton LoF variants' distribution using selected variants between human NTDs and 1KGP.

Gene set of 249 human orthologs of mouse NTD associated genes, which have been identified based on mouse NTD model studies[13], was first used to investigate the enrichment of these rare damaging variants.

Singleton LoF variant confirmation

120 SLoFVs were randomly selected in Chinese NTDs cohort and confirmed by Sanger sequencing. 88.3% (106/120) were correct. Also 28 SLoFVs were randomly selected in US NTDs of which 96.4% (27/28) were confirmed by Sanger sequencing.

Pathway analysis

Frequently mutated pathways with rare LoF variants in human NTDs were analyzed using KEGG pathway[14,15] via R packages[16,17]. Heat-map was generated by the Complex Heat-map package in R[18] to determine the relationship between samples and pathways with LoF variants.

Genome expression and DNA methylation analysis

The genome expression profile of GSE4182, which included fetal mRNA data of 4 NTDs and 5 controls based on oligonucleotide microarrays, was downloaded from the Gene Expression Omnibus database[19]. Surrogate variable analysis (SVA) was applied to remove batch effects[20]. Gene annotation was added by hgu133plus2.db package in R. We used the limma package[21] to screen the differentially expressed genes between cases and controls. The *P*-values were adjusted for multiple comparisons using the FDR (False Discovery Rate). The adjusted $P < 0.05$ and 1.2-fold were used as the cut-off criteria. Function heatmap.2 was used for the graphical display of the dendrogram[22]. We used DOSE package[23] for pathway enrichment analysis.

Statistical analysis

Differential distributions of LoF variants between human NTDs and 1KGP were tested by Wilcoxon rank sum test. The χ^2 and Fisher's exact test were employed for association analysis of pathways with LoF variants in human NTDs, and the *P*-values were adjusted using the Bonferroni correction. Statistical analyses were conducted by R (http://cran.r-project.org).

References

1 Genomes Project, C. *et al.* A global reference for human genetic variation. *Nature* **526**, 68-74, doi:10.1038/nature15393 (2015).

2 Sportoletti, P. *et al.* NOTCH1 PEST domain mutation is an adverse prognostic factor in B-CLL. *Br J Haematol* **151**, 404-406, doi:10.1111/j.1365-2141.2010.08368.x (2010).

3 McKenna, A. *et al.* The Genome Analysis Toolkit: a MapReduce framework for analyzing next-generation DNA sequencing data. *Genome research* **20**, 1297-1303, doi:10.1101/gr.107524.110 (2010).

4 DePristo, M. A. *et al.* A framework for variation discovery and genotyping using next-generation DNA sequencing data. *Nature genetics* **43**, 491-498, doi:10.1038/ng.806 (2011).

5 Van der Auwera, G. A. *et al.* From FastQ data to high confidence variant calls: the Genome Analysis Toolkit best practices pipeline. *Current protocols in bioinformatics* **43**, 11 10 11-33, doi:10.1002/0471250953.bi1110s43 (2013).

6 Zhao, H. *et al.* CrossMap: a versatile tool for coordinate conversion between genome assemblies. *Bioinformatics* **30**, 1006-1007, doi:10.1093/bioinformatics/btt730 (2014).

7 McLaren, W. *et al.* Deriving the consequences of genomic variants with the Ensembl API and SNP Effect Predictor. *Bioinformatics* **26**, 2069-2070, doi:10.1093/bioinformatics/btq330 (2010).

8 Rodriguez, J. M. *et al.* APPRIS 2017: principal isoforms for multiple gene sets. *Nucleic acids research*, doi:10.1093/nar/gkx997 (2017).

9 Sveinbjornsson, G. *et al.* Weighting sequence variants based on their annotation increases power of whole-genome association studies. *Nat Genet* **48**, 314-317, doi:10.1038/ng.3507 (2016).

10 Eilbeck, K. *et al.* The Sequence Ontology: a tool for the unification of genome annotations. *Genome biology* **6**, R44, doi:10.1186/gb-2005-6-5-r44 (2005).

11 Adzhubei, I. A. *et al.* A method and server for predicting damaging missense mutations. *Nature methods* **7**, 248-249, doi:10.1038/nmeth0410-248 (2010).

12 Kumar, P., Henikoff, S. & Ng, P. C. Predicting the effects of coding non-synonymous variants on protein function using the SIFT algorithm. *Nature protocols* **4**, 1073-1081, doi:10.1038/nprot.2009.86 (2009).

13 Harris, M. J. & Juriloff, D. M. An update to the list of mouse mutants with neural tube closure defects and advances toward a complete genetic perspective of neural tube closure. *Birth defects research. Part A, Clinical and molecular teratology* **88**, 653-669, doi:10.1002/bdra.20676 (2010).

14 Kanehisa, M., Sato, Y., Kawashima, M., Furumichi, M. & Tanabe, M. KEGG as a reference resource for gene and protein annotation. *Nucleic acids research* **44**, D457-462, doi:10.1093/nar/gkv1070 (2016).

15 Kanehisa, M., Furumichi, M., Tanabe, M., Sato, Y. & Morishima, K. KEGG: new perspectives on genomes, pathways, diseases and drugs. *Nucleic acids research* **45**, D353-D361, doi:10.1093/nar/gkw1092 (2017).

16 Luo, W. & Brouwer, C. Pathview: an R/Bioconductor package for pathway-based data integration and visualization. *Bioinformatics* **29**, 1830-1831, doi:10.1093/bioinformatics/btt285 (2013).

17 Yu, G., Wang, L. G., Han, Y. & He, Q. Y. clusterProfiler: an R package for comparing biological themes among gene clusters. *OMICS* **16**, 284-287, doi:10.1089/omi.2011.0118 (2012).

18 Gu, Z., Eils, R. & Schlesner, M. Complex heatmaps reveal patterns and correlations in multidimensional genomic data. *Bioinformatics* **32**, 2847-2849, doi:10.1093/bioinformatics/btw313 (2016).

19 Nagy, G. R. *et al.* Use of routinely collected amniotic fluid for whole-genome expression analysis of polygenic disorders. *Clin Chem* **52**, 2013-2020, doi:10.1373/clinchem.2006.074971 (2006).

20 Leek, J. T., Johnson, W. E., Parker, H. S., Jaffe, A. E. & Storey, J. D. The sva package for removing batch effects and other unwanted variation in high-throughput experiments. *Bioinformatics* **28**, 882-883, doi:10.1093/bioinformatics/bts034 (2012).

21 Smyth, G. K. Linear models and empirical bayes methods for assessing differential expression in microarray experiments. *Stat Appl Genet Mol Biol* **3**, Article3, doi:10.2202/1544-6115.1027 (2004).

22 Gentleman, R. C. *et al.* Bioconductor: open software development for computational biology and bioinformatics. *Genome biology* **5**, R80, doi:10.1186/gb-2004-5-10-r80 (2004).

23 Yu, G., Wang, L. G., Yan, G. R. & He, Q. Y. DOSE: an R/Bioconductor package for disease ontology semantic and enrichment analysis. *Bioinformatics* **31**, 608-609, doi:10.1093/bioinformatics/btu684 (2015).
