# Supplementary material for: Threshold for neural tube defect risk by accumulated singleton loss-of-function variants
Source: Cell Res. 2018 Jul 5;28(10):1039–41. doi: 10.1038/s41422-018-0061-3 (PMC6170406; doi:10.1038/s41422-018-0061-3)
Supplement: Supplementary file 2 — Supplementary information, Tables S1-S4 [file 41422_2018_61_MOESM2_ESM.pdf]

Table S1. Demographic characteristics in three different NTD cohorts.

Variable	China Cohort	US Cohort	ME Cohort
Case #	100	74	69
Region	Shanxi	Texas	Qatar
Age	20.7 ± 4.7(weeks)	7.5 ± 5.4 (years)	7.23 (years)
Gender:			
Male	60 (60%)	36 (48.6%)	28 (40.6%)
Female	37 (37%)	38 (51.4%)	41 (59.4%)
Unknown	3 (3%)	0	0
AE	95 (95%)	0	0
CRS	66 (66%)	0	0
OEC	33 (33%)	0	0
MM	1 (1%)	100%	100%

AE, anencephaly; **CRS**, craniorachischisis; O**EC**, occipital encephalocele; **MM**, myelomeningocele.

Table S2. The numbers of carriers with damaging variants were compared between Han Chinese NTDs and controls. Only damaging variants in the human orthologs of 249 previously reported NTD-associated genes in mouse were counted here (gene list was extracted from https://ntdwiki.wikispaces.com).

Type	carriers (no./total no.)		*P* Value[a]
	NTD patients	1 KGP	
LoF	7/100	13/208	1
D-mis	56/100	141/208	0.39
LoF+D-mis	60/100	146/208	0.48

[a]*P* values were estimated using χ^2 test.

Table S3 Number of carriers of SLoFVs among NTDs and controls.

Race	Number of SLoFVs		P [a]
China Cohort	Cases, n = 100	Controls (CHB&CHS), n = 208	7.84×10^{-114}
	1500	1318	
US Cohort	Cases, n = 74	Controls (CEU), n = 99	2.03×10^{-39}
	834	547	
ME Cohort	Cases, n = 69	Controls (1KGP), n = 2504	1.45×10^{-9}
	516	14107	
All NTDs	Cases, n = 243	Controls (1KGP), n = 2504	5.97×10^{-237}
	2850	14107	

[a] *P* values were estimated using binomial test.

Table S4 SLoFV enrichment analysis in the pathways with most SLoFVs.

Pathway name	CDS length [a]	#NTD/Ctrl [b]	P_{value} [c]	P_{fdr} [d]	P_{bonf} [e]
Metabolic pathways	2048017	84/109	0.092	0.29	1
Olfactory transduction	523344	35/28	0.0046	0.059	1
Pathways in cancer	815210	25/19	0.011	0.089	1
PI3K-Akt signaling pathway	787584	24/23	0.064	0.212	1
Neuroactive ligand-receptor interaction	440236	22/21	0.075	0.246	1
Tight junction	385998	22/14	0.0052	0.059	1
Focal adhesion	649757	21/22	0.139	0.323	1
Vascular smooth muscle contraction	270444	20/9	0.0009	0.042	0.252
Huntington's disease	421947	20/23	0.238	0.496	1
cGMP-PKG signaling pathway	366986	19/9	0.0016	0.053	0.469
Oxytocin signaling pathway	356229	19/8	0.0008	0.042	0.232
cAMP signaling pathway	456229	18/7	0.0007	0.042	0.209
Inflammatory mediator regulation of TRP channels	212076	18/6	0.00031	0.03	0.091
Bile secretion	158019	18/10	0.0054	0.059	1

[a] Length of coding sequences in corresponding pathway.

[b] Number of SLoFV in NTD cohorts (174 Chinese Han and US CEU) and control (307 Chinese Han and CEU genomes from the 1KGP).

[c] *P* values were estimated using χ^2 test.

[d] *P* values were adjusted with FDR method.

[e] *P* values were adjusted with Bonferroni correction.
