# Supplementary material for: Threshold for neural tube defect risk by accumulated singleton loss-of-function variants
Source: Cell Res. 2018 Jul 5;28(10):1039–41. doi: 10.1038/s41422-018-0061-3 (PMC6170406; doi:10.1038/s41422-018-0061-3)
Supplement: Supplementary file 3 — Supplementary information, Figures S1 and S2 [file 41422_2018_61_MOESM3_ESM.pdf]

Supplementary information, Figure S1

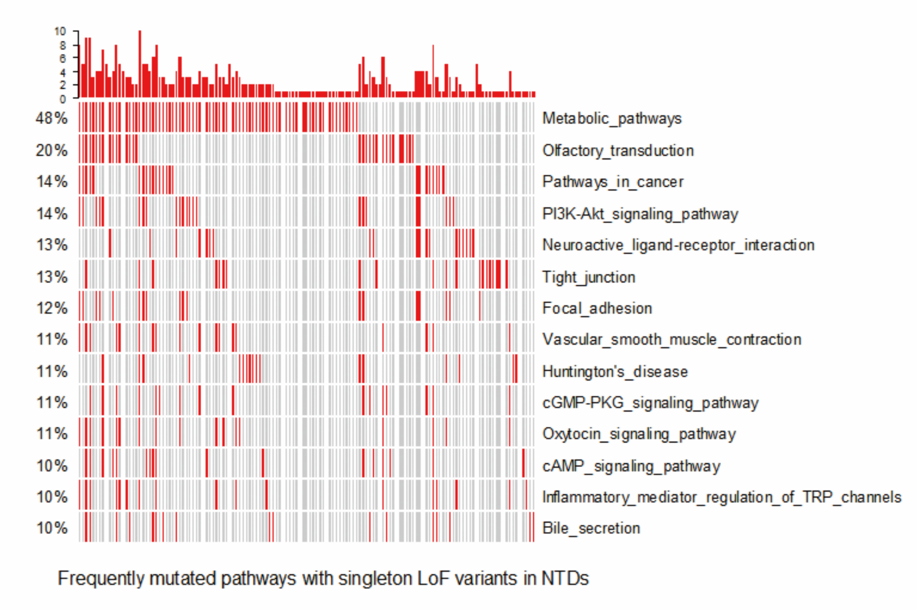

Figure S1 Frequently mutated pathways with SLoFVs in NTDs (including Chinese and US NTDs). Pathways are shown according to decreasing SLoFV frequency. NTD samples are displayed as columns. Left bar depicts the percentage of NTD samples with SLoFVs.

Supplementary information, Figure S2

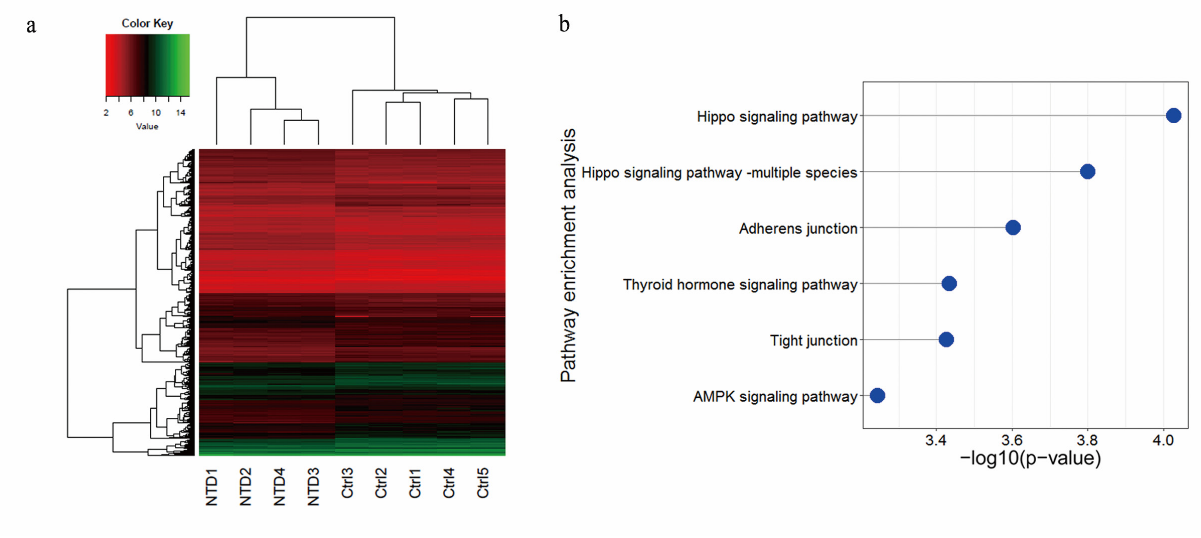

Figure S2 Hierarchical clustering and KEGG pathway enrichment analysis based on significantly differentially expressed genes ($P < 0.05$ and 1.2-fold change) of NTDs and controls. **a** Hierarchical clustering analysis of significantly differentially expressed genes in NTDs and controls based on GSE4182. **b** KEGG pathway enrichment analysis based on significantly differentially expressed genes of NTDs and controls.
